# Corneal Infection Models: Tools to Investigate the Role of Biofilms in Bacterial Keratitis

**DOI:** 10.3390/cells9112450

**Published:** 2020-11-10

**Authors:** Lucy Urwin, Katarzyna Okurowska, Grace Crowther, Sanhita Roy, Prashant Garg, Esther Karunakaran, Sheila MacNeil, Lynda J. Partridge, Luke R. Green, Peter N. Monk

**Affiliations:** 1 Department of Infection, Immunity and Cardiovascular Disease, University of Sheffield, Sheffield S10 2RX, UK; l.r.green@sheffield.ac.uk (L.R.G.); p.monk@sheffield.ac.uk (P.N.M.); 2 Department of Chemical and Biological Engineering, University of Sheffield, Sheffield S1 3JD, UK; k.emery@sheffield.ac.uk (K.O.); gracescrowther@gmail.com (G.C.); e.karunakaran@sheffield.ac.uk (E.K.); 3 Sheffield Collaboratorium for Antimicrobial Resistance and Biofilms (SCARAB), University of Sheffield, Sheffield S1 3JD, UK; s.macneil@sheffield.ac.uk (S.M.); l.partridge@sheffield.ac.uk (L.J.P.); 4 Prof. Brien Holden Eye Research Centre, LV Prasad Eye Institute, Hyderabad 500034, India; sanhita@lvpei.org (S.R.); prashant@lvpei.org (P.G.); 5 Department of Materials Science and Engineering, University of Sheffield, Sheffield S1 3JD, UK; 6 Department of Molecular Biology and Biotechnology, University of Sheffield, Sheffield S10 2TN, UK

**Keywords:** microbial keratitis, bacterial keratitis, cornea, infection, biofilm, models, *in vitro*, *ex vivo*, *in vivo*

## Abstract

Bacterial keratitis is a corneal infection which may cause visual impairment or even loss of the infected eye. It remains a major cause of blindness in the developing world. *Staphylococcus aureus* and *Pseudomonas aeruginosa* are common causative agents and these bacterial species are known to colonise the corneal surface as biofilm populations. Biofilms are complex bacterial communities encased in an extracellular polymeric matrix and are notoriously difficult to eradicate once established. Biofilm bacteria exhibit different phenotypic characteristics from their planktonic counterparts, including an increased resistance to antibiotics and the host immune response. Therefore, understanding the role of biofilms will be essential in the development of new ophthalmic antimicrobials. A brief overview of biofilm-specific resistance mechanisms is provided, but this is a highly multifactorial and rapidly expanding field that warrants further research. Progression in this field is dependent on the development of suitable biofilm models that acknowledge the complexity of the ocular environment. Abiotic models of biofilm formation (where biofilms are studied on non-living surfaces) currently dominate the literature, but co-culture infection models are beginning to emerge. *In vitro*, *ex vivo* and *in vivo* corneal infection models have now been reported which use a variety of different experimental techniques and animal models. In this review, we will discuss existing corneal infection models and their application in the study of biofilms and host-pathogen interactions at the corneal surface.

## 1. Introduction

Bacterial keratitis is a potentially sight-threatening eye infection, localised to the cornea. The infection is characterised by the presence of replicating bacteria on the ocular surface, which disrupt the integrity of the corneal epithelium and result in inflammation of the corneal stroma [1]. Early symptoms include pain, redness, excessive lacrimation, light sensitivity and blurred vision. Examination of the eye reveals lid oedema, congestion of conjunctiva, corneal haze and a variable degree of inflammation of the anterior chamber. The condition results in corneal scarring or in extreme cases, corneal perforation and loss of the eye [2]. According to the World Health Organization, corneal blindness is currently the fourth largest contributor to global blindness [3] and instances of ocular trauma/corneal ulceration have been estimated to cause 1.5-2 million new cases of monocular blindness per year [4]. Following ocular surface trauma, the cornea becomes highly susceptible to infection and so many of these cases involve an infection component. Corneal infections may be caused by bacteria, fungi, viruses or protozoans (collectively termed ‘microbial keratitis’) [5] but this review will focus solely on bacterial keratitis. Bacterial species most commonly responsible for bacterial keratitis include *Staphylococcus aureus* and *Pseudomonas aeruginosa*, although geographic variations in predominance have been reported [6,7,8].

### Predisposing Risk Factors

The healthy cornea is highly resistant to infection and so microbial keratitis rarely occurs in the absence of predisposing risk factors [9]. Reported risk factors include corneal trauma, contact lens-wear, chronic ocular surface disease, ocular surgery and systemic diseases associated with an immunocompromised state [10,11]. These factors compromise the resistance mechanisms employed by the cornea, rendering it newly susceptible to infection [9]. Although microbial keratitis occurs in both developed and developing countries, there are large differences in the disease epidemiology and aetiology [12]. In developing countries, corneal trauma constitutes the major risk factor in the development of microbial keratitis and this is thought to reflect the increased size of the agricultural workforce in these countries, e.g., rice stalks and thorns are a common cause of ocular injury for farmers in South India [13,14]. Support for this is provided by a recent epidemiological study, conducted in South India (n = 252). Ocular trauma was reported for 72% of microbial keratitis infections and 63% of patients were employed as agriculturists [15]. In contrast, contact lens-wear constitutes the major risk factor in the development of microbial keratitis in more developed countries. Studies conducted in France and Sweden identified contact-lens wear as the major risk factor in ~50% of cases [10,16] and steep rises in the incidence of microbial keratitis in developed countries have been linked to the increased popularity of contact lenses. In Minnesota, a 435% increase in microbial keratitis was recorded over a 39-year period, following the introduction of contact-lenses [17].

## 2. Biofilms

**Figure 1:**
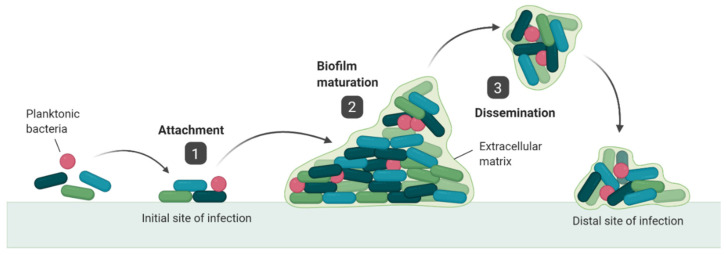
The biofilm life-cycle. The biofilm life-cycle consists of three main stages: (1) Attachment: Planktonic, free-living bacteria adhere to a surface. This is mediated by a combination of physical factors (e.g., surface hydrophobicity, electrostatic interactions) and bacterial appendages (e.g., pili, flagella). (2) Biofilm maturation: Adherent bacteria proliferate to form microcolonies. Bacteria produce Extracellular Polymeric Substances (EPS), EPS matrix stabilises the bacterial network. (3) Dissemination: A subset of bacteria detach, regain some of the characteristics of planktonic bacteria and colonise distal sites.

A biofilm has been defined as “a microbially derived sessile community characterized by cells that are irreversibly attached to a substratum or interface or to each other, are embedded in a matrix of extracellular polymeric substances that they have produced, and exhibit an altered phenotype with respect to growth rate and gene transcription” [18]. Biofilms form on both biotic and abiotic surfaces and are ubiquitous in infection. It is estimated that over 80% of microbial infections affecting the human body involve a biofilm component [19], and this includes various ocular infections [20,21]. Biofilm formation has been directly visualised at the corneal surface during experimental corneal infection [22] and bacteria obtained from corneal infections display biofilm forming activity [23,24]. The biofilm life-cycle (Figure 1) can be divided into three main stages: Initial attachment, biofilm maturation and dissemination [25]. The process of dissemination creates particular challenges in the treatment of biofilm infections, as it allows biofilm bacteria to regain their planktonic characteristics and colonise distal sites within the body. As a result, many biofilm infections become chronic and are recurrent in nature [26] and eradication of biofilms is further complicated by an enhanced resistance phenotype [27,28,29]. Biofilm literature is currently dominated by abiotic models which lack any living cells [30]. Numerous studies have investigated biofilm formation on contact lenses/lens cases, as well as the efficacy with which different lens materials and disinfecting solutions can be used to reduce bioburden [31,32,33,34,35]. However, these models lack crucial interaction between bacteria and animal/human tissues. To understand various aspects of biofilm and their true implications it will be important that co-culture models investigating biofilm formation at the biotic surface are developed. In this review, we will focus on the presence of biofilm at the biotic corneal surface.

## 3. Antibiotic Resistance in Biofilms

A major concern for the treatment of bacterial keratitis is the emergence of antibiotic resistance. Two of the most common causative agents, *S. aureus* and *P. aeruginosa*, are ESKAPE pathogens: an acronym used by the Infectious Diseases Society of America to describe 6 major pathogens (*Enterococcus faecium, Staphylococcus aureus, Klebsiella pneumoniae, Acinetobacter baumannii, Pseudomonas aeruginosa*, and *Enterobacter* species) that commonly cause nosocomial infections and that use various antibiotic resistance mechanisms [36,37]. Furthermore, *S. aureus* and *P. aeruginosa* have been identified by the World Health Organization as ‘High’ and ‘Critical’ priority targets, in the development of new antibiotics [38]. Evidence of increasing antibiotic resistance among ocular isolates is provided by longitudinal studies [39,40] and of particular concern is the observation that resistance to fluoroquinolones is increasing for both Methicillin Susceptible (MSSA) and Methicillin Resistant (MRSA) *S. aureus* strains [40]. In the UK, fluoroquinolones are currently used as the first-line antibiotic in the treatment of bacterial keratitis [41]. Antibiotic resistance data for *P. aeruginosa* ocular isolates is varied, with studies reporting Multi-Drug Resistant (MDR) isolates in the range of 6.52-42.9% [42,43]. Overall, the US Department of Health and Human Services has reported that MDR *P. aeruginosa* constitutes 13% of all *P. aeruginosa* infections (n = 6700) [44]. Infections involving antibiotic resistant bacteria are difficult to treat and often require combination antibiotics in high doses (fortified therapy) instead of the standard fluoroquinolone monotherapy. Furthermore, increased levels of antibiotic resistance have been linked to poorer clinical outcomes, with one study reporting a significant association between the Minimum Inhibitory Concentration (MIC) of the treatment antibiotic(s) and the length of healing time for corneal ulcers [45].

### 3.1. Mechanisms of Biofilm-Specific Antibiotic Resistance

To tackle the growing threat of antibiotic resistance, it is important that we understand the antibiotic resistance mechanisms used by bacteria. Resistance is largely attributable to genetic mutations and the acquisition of specific antibiotic resistance genes by horizontal gene transfer. Common genetic mechanisms include the presence/overexpression of efflux pumps that remove antibiotics, stimulation of modifying enzymes that inactivate antibiotics and the modification of bacterial target sites [46]. The formation of biofilms has also been associated with an increased resistance to antimicrobials [27]. However, biofilm-specific antimicrobial resistance does not appear to be governed by the same genetic elements that confer resistance in planktonic bacteria and dissemination of bacterial biofilms has been associated with a return in susceptibility to antimicrobials [47]. This suggests that the multicellular nature of biofilms is central in explaining biofilm-specific antibiotic resistance, a topic more extensively reviewed elsewhere [48,49] and three main hypotheses that acknowledge the importance of multicellularity have been proposed.

#### 3.1.1. Limited Antimicrobial Penetration

One explanation is that the presence of a biofilm network limits antimicrobial penetration, thereby preventing effective concentrations of antibiotic from reaching all of the cells in the population. Not only does the presence of a biofilm act as a physical barrier, but antimicrobials may be deactivated or sequestered within the extracellular matrix environment [50]. For instance, alginate, eDNA and periplasmic glucans are all components of the *P. aeruginosa* biofilm environment that have been shown to impede the movement of antibiotics [51,52]. Further support is provided by a recent study investigating the relationship between antibiotic penetration and bacterial clearance. Antibiotics that diffused more readily across *S. aureus* biofilms were associated with decreased bacterial recovery, demonstrating that the ability to penetrate the biofilm matrix is important in reducing bacterial load [53]. However, there is one major problem with this explanation, which is that if transport limitations were a major factor in antibiotic resistance, we would expect the effectiveness of antibiotics to return once the biofilm matrix became saturated with the drug compound. On the contrary, biofilm bacteria have been shown to exhibit continued resistance at antibiotic concentrations that greatly exceed the minimum inhibitory concentration (MIC) and minimum bactericidal concentration (MBC) [54,55].

#### 3.1.2. The Presence of Altered Chemical Microenvironments

Further support for the idea that transport limitation does not play a major role in antimicrobial resistance is provided by a study investigating the relative effects of antibiotic penetration, metabolic activity and oxygen availability on the antibiotic resistant phenotype of *P. aeruginosa* biofilms [56]. The use of a diffusion bioassay revealed that both ciprofloxacin and tobramycin are able to penetrate *P. aeruginosa* biofilms and that zones displaying greatest antibiotic resistance corresponded with areas of low metabolic activity and oxygen concentration, rather than decreased antibiotic concentration. These findings provide support for an alternative explanation, in which increased resistance to antimicrobials is attributable to the presence of altered microenvironments within biofilms. These microenvironments include areas of waste product build up, altered pH and anaerobic niches that cause antimicrobials to work less effectively than they would in the bulk environment [57,58]. The presence of these altered chemical environments not only interferes with the activity of antimicrobials but also causes metabolic heterogeneity to arise within biofilms. This heterogeneity makes it difficult for one single antimicrobial to target and kill all of the members of the bacterial population.

#### 3.1.3. Persister Cells

Another source of heterogeneity and a third explanation for biofilm-specific resistance is the existence of phenotypic variants named ‘persisters’. This subset of cells constitutes a very small proportion of the total bacterial population (~1%), but the presence of persisters has been documented since the mid-1900s [59]. A recent Nature review article defined these cells as “a subpopulation of transiently antibiotic-tolerant bacterial cells that are often slow-growing or growth-arrested, and are able to resume growth after a lethal stress” [60]. The transcription of genes involved in energy production is downregulated in persister cells [61] and a reduced growth phenotype exists prior to antibiotic treatment [62]. Since many antibiotics work by targeting metabolic pathways, it has been hypothesised that persister cells evade the bactericidal effects of antibiotics as a result of their inactivity [60].

## 4. Immune Evasion in Biofilms

During bacterial infections, host cell Pattern Recognition Receptors (PRRs) detect invading pathogens via the recognition of Pathogen Associated Molecular Patterns (PAMPs). This stimulates an innate immune response involving the activation of complement and various chemotactic signalling pathways, which allow immune cells to be recruited to the site of infection [63,64]. Innate immune cells such as polymorphonuclear neutrophils (PMNs) and macrophages are then able to kill invading bacteria via phagocytosis and the production of bactericidal compounds (e.g., elastase enzymes, lactoferrin and reactive oxygen species) [65]. When challenged by planktonic bacteria, the innate immune response is highly efficient in its clearance of bacteria but the presence of bacterial biofilms has been associated with an increased resistance to host defences [66,67]. Mechanisms of biofilm-specific immune evasion include mechanical protection, shielding from immune recognition, changes in gene expression and inhibition of immune cell functions [68,69,70]. It is important to note that there are differences in the immune evasion strategies used by different bacterial species (heavily influenced by EPS composition), but this review is focused solely on *S. aureus* and *P. aeruginosa*.

### 4.1. Role of Extracellular Polymeric Substances (EPS)

#### 4.1.1. Mechanical Protection

Common to all bacterial biofilms, is the presence of an EPS matrix that encases the bacterial cells. This matrix is important in aggregating the individual cells together and in doing so creates a formidable barrier against phagocytosis. This killing mechanism is dependent on the engulfment of bacteria and so phagocytes are only able to phagocytose bacterial targets up to their own cell size. Therefore, when bacteria are aggregated together as biofilm populations, a dysfunction in immune-cell-killing, termed “frustrated phagocytosis”, is observed [71,72]. In PMNs (cell size ~10 μm), frustrated phagocytosis is observed at a polystyrene bead diameter of 11.2 μm, with only 50% engulfment of bead circumference [73]. To overcome this size barrier, biofilm structures must be broken apart. PMNs are predicted to exert attractive stresses of up to 1kPa that could facilitate biofilm disruption. However, EPS matrix composition has been shown to influence resistance to mechanical attack and in *P. aeruginosa*, increased expression of the Psl polysaccharide is associated with increases in biofilm stiffness and intercellular cohesion between bacterial cells [74]. This led the authors to suggest that changes in biofilm mechanics could allow biofilms to resist PMN stress, or at least delay biofilm disruption long enough for bacterial PMN killing mechanisms to take effect.

#### 4.1.2. Immune Recognition

As well as providing mechanical protection against phagocytosis, components of the EPS also help to prevent immune recognition, thereby reducing phagocytic clearance. For instance, coagulase expression causes fibrin to accumulate in the EPS environment of *S. aureus* biofilms [75], this fibrous protein is known to protect staphylococci from opsonophagocytic killing [76]. In other staphylococcal strains, polymeric-N-acetyl-glucosamine (PNAG) has been described as an antibody “sink” and is shown to protect against the binding of IgG and C3b to biofilm-bacteria [77,78]. Although this has not been demonstrated in *S. aureus*, it is possible that similar principles could apply, with EPS components acting as decoys for opsonisation and/or preventing direct targeting of the biofilm bacteria [70]. In *P. aeruginosa*, alginate and Psl polysaccharide are major components of the EPS. The presence of alginate has been shown to reduce both opsonic and non-opsonic phagocytosis [29,79], with protection against opsonic antibodies mediated by O-acetylation of alginate [80]. Similarly, the presence of Psl has been shown to reduce opsonic phagocytosis but via disruption of complement binding [81].

### 4.2. Changes in Gene Expression

The upregulation of genes encoding toxins and immune evasion proteins plays a major role in the increased immune resistance of *S. aureus* biofilms. Upregulated toxins include Hla, LukAB/GH, LukED, HlgAB, HlgCB and PSMs and upregulated immune evasion proteins include Eap, CHIPS, SAK and SSL10 [70]. Gene expression is controlled by the accessory gene regulator (Agr) quorum sensing (QS) system [82]. Interestingly, Agr dysfunction is common within *S. aureus* biofilm populations, whereas there is a strong selective pressure against QS mutations in planktonic bacteria [83]. Agr controls the expression of several protease enzymes involved in biofilm dispersal and so Agr mutants form thick, dense biofilms [84,85] with an increased resistance to antibiotics [86]. Therefore, it has been suggested that Agr heterogeneity may represent another biofilm specific host defence mechanism; the Agr functional bacteria produce toxins that actively target host immune cells while Agr dysfunctional bacteria strengthen the mechanical barrier against phagocytes [83,87]. *P. aeruginosa* bacteria also undergo significant changes in gene expression following the switch to a biofilm lifestyle. Following initial adhesion, there is a large increase in intracellular cyclic diguanylate monophosphate (c-di-GMP) concentration [88]. This second messenger molecule controls the expression of over 500 genes, including genes implicated in immune evasion [88,89]. For instance, c-di-GMP signalling induces a switch from flagellar to twitching motility and downregulates the expression of PAMPs including the flagellum and PcrV [90]. This prevents activation of the host NAIP/NLRC4 inflammasome and limits phagocyte recruitment [91,92]. However, the importance of the host inflammasome in resolving *P. aeruginosa* infections has been challenged [90].

### 4.3. Manipulation of Host Immune Cells

Despite the presence of the EPS, innate immune cells have been shown to penetrate both *S. aureus* and *P. aeruginosa* biofilms [93,94]. However, this does not guarantee bacterial clearance, and within this environment, immune cells are exposed to various host-killing mechanisms. For instance, *P. aeruginosa* biofilms produce N-3-oxododecanoyl homoserine lactone (3-oxo-C12-HSL), a QS molecule that induces apoptosis of both PMNs and macrophages [95]. Similarly, rhamnolipid biosynthesis has been linked to PMN lysis in *P. aeruginosa* biofilms [96] and the production of various pore-forming leukocidins is responsible for leukocyte cell death in *S. aureus* infections [97]. PMN lysis not only prevents phagocytosis, but also enhances the formation of *P. aeruginosa* biofilms as biofilm bacteria can incorporate host DNA and actin protein into their own EPS environment [98]. This increases biofilm matrix stability and resistance to antimicrobials [99]. The biofilm-enhancing effects of PMN lysis have been demonstrated in bacterial keratitis, with eDNA/F-actin acting as cellular scaffolds to promote bacterial colonization [100]. Another way in which biofilm bacteria exploit host immune cells is via their interactions with Neutrophil Extracellular Traps (NETs). NETs are formed of host DNA and granule proteins and are produced by PMNs in response to infection [101]. They bind to and aggregate invading bacteria, targeting them for host cell destruction. NETs play a particularly important role in *P. aeruginosa* keratitis, preventing the spread of bacteria to the brain [102]. *S. aureus* circumvents NET activity via the production of nuclease and adenosine synthase enzymes, which degrade the NET DNA into deoxyadenosine (dAdo) [103,104]. This molecule is pro-apoptotic and causes the caspase-3-mediated cell death of PMNs and macrophages [105], thereby preventing the phagocytic clearance of *S. aureus* biofilms. Furthermore, biofilm bacteria are able to modulate immune cell behaviour via the production of second messenger molecules such as cyclic diadenylate monophosphate (c-di-AMP). C-di-AMP stimulates a type I interferon response in host cell macrophages and this is associated with an anti-inflammatory M2 phenotype that facilitates the intracellular survival of *S. aureus* [106]. This is an example of immune polarization, a phenomenon that is becoming increasingly recognised in the persistent nature of biofilm infections. Various immune cells are able to adopt distinct phenotypes (e.g., M1 vs M2 macrophages) and it appears pathogenic bacteria (including *S. aureus* and *P. aeruginosa*) are able to skew the immune response towards a balance that facilitates chronic infection [107].

## 5. Modelling Biofilm Infections

Bacteria have been shown to colonise the cornea as biofilm populations during bacterial keratitis. To study corneal biofilm infections in a meaningful way, it is important that biofilm models are representative of the true infectious scenario. Since abiotic models do not allow host–pathogen interactions to be studied, they are unsuitable for many elements of infection research. Fortunately, several biotic biofilm models that use *in vitro*, *ex vivo* or *in vivo* corneal systems have been developed. However, there are various advantages and disadvantages associated with each of these models that must be carefully considered (Table 1).

### 5.1. In Vitro Models

**Figure 2:**
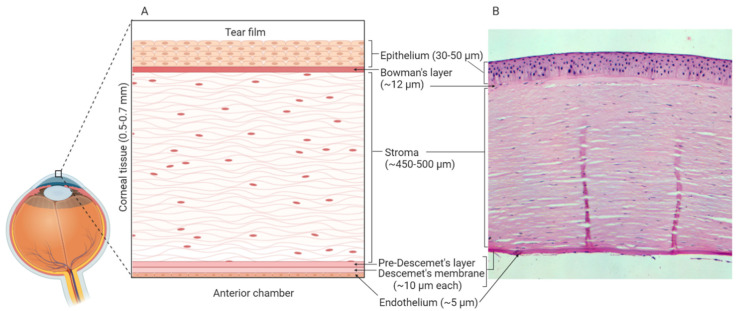
Human corneal layers: (**A**) Schematic representation, and (**B**) haematoxylin and eosin staining. The cornea has six distinct layers and the outermost layer is the corneal epithelium, which is made up of 5-7 rows of tightly packed corneal epithelial cells. These cells lie on an acellular, collagenous layer named the Bowman’s layer and together the epithelium and the Bowman’s layer are essential in the protection of the underlying stromal tissue. The stroma constitutes 90% of the overall thickness of the cornea and is composed of mainly type I collagen and differentiated keratocytes. Beneath the stroma is the Pre-Descemet’s layer (also known as Dua’s layer) and the Descemet’s membrane. These collagen-rich, acellular layers separate the stromal tissue from the endothelium. The endothelium is composed of a single layer of cells, which are mainly hexagonal in shape. This layer is adjacent to the anterior chamber and constitutes the final layer of the cornea. Created with Biorender.com.

*In vitro* models use well-defined cell culture techniques to generate 3D corneal constructs. These models are a popular choice for ophthalmological research due to their relative cost-effectiveness and limited use of animals. The human cornea is composed of six distinct layers: epithelium, Bowman’s layer, stroma, Pre-Descemet’s layer, Descemet’s membrane and endothelium [108,109] (Figure 2). As the outermost layer, the corneal epithelium constitutes the first line of defence against external pathogens and also acts as the major barrier against ocular drug penetration [110]. Therefore, many *in vitro* models have focused solely on the cultivation of human corneal epithelial cell (HCE) multilayers [111,112]. However, 3D organotypic models have also been developed which incorporate epithelial, stromal and endothelial cells, providing whole-tissue models [113,114,115].

Another source of model diversity is the use of primary cells versus immortalized cell lines. Primary cells are extracted directly from donor corneal tissue and therefore share the same phenotypic and genotypic characteristics as the donor tissue. The drawback is that these cells have a finite lifespan and reach senescence after only a few passages [116]. Furthermore, the availability of human corneal tissue is highly limited as healthy tissue is generally reserved for keratoplasty. This means animal corneas are often used as a source of primary corneal epithelial cells.

The production of an immortalized cell line involves transfection/transformation of cells with a virus or plasmid that induces the cells to enter a continuously growing state by activating telomere maintenance mechanisms [117]. As a result, the cells may be continuously passaged, and cell lines are commercially available. This makes immortalized cell lines attractive model systems, as they are easy to assemble and economical. However, the underlying assumption that cell lines mimic all aspects of the normal cornea has not been proven and with each passage, genetic drift occurs, causing cells to become phenotypically distinct from the original cell population [118]. A study comparing the gene expression profile of the HCE-T cell line to gene expression in the healthy human cornea, found changes in gene expression for 36% of probed genes [119]. This is a reminder of the importance of characterising cell lines to ensure they remain suitably representative of the ocular surface *in vivo*.

#### Existing *In Vitro* Infection Models

Drug permeation studies have been a key driver in the development of *in vitro* corneal models. Curved filters have been used to produce monolayers that share the curvature of the cornea [120] and optimisation of cell culture conditions has led to the development of corneal models with tight cell junctions, epithelial barrier integrity and permeation profiles comparable to those of the excised cornea [121,122]. The development of *in vitro* models for studying corneal absorption has been reviewed previously [123,124] and optimised cell culture techniques are transferable to the development of *in vitro* infection models. Such models have been used to investigate host–pathogen interactions at the corneal epithelial surface. Immortalized HCE cell lines have been used to investigate receptor-mediated adhesion mechanisms and identify key bacterial virulence factors (VFs) involved in invasion [125,126]. Modulation of the host response has also been studied, with a recent study demonstrating that the type-III secretion system (T3SS) of *P. aeruginosa* is involved in subversion of antimicrobial peptide (AMP) expression [127]. Furthermore, *in vitro* studies have demonstrated the importance of host cell defences such as cell surface mucins and tear fluid. Knockdown of MUC16 in the HCLE cell line causes significant decreases in epithelial barrier function [128] and exposure of primary rabbit corneal epithelial cells to human tear fluid has been shown to confer significant cytoprotective effects, as well as reducing the translocation of *P. aeruginosa* [129,130]. These *in vitro* infection models have helped to progress our understanding of bacterial keratitis, but they are limited by the absence of a biofilm component. To the best of our knowledge, an *in vitro* model that combines live HCE cells and the formation of bacterial biofilm is yet to be reported. In contrast, multiple keratitis studies have investigated biofilm formation on abiotic surfaces in the absence of cells [131,132]. As *in vitro* modelling techniques continue to improve, co-culture models may be reported but there are various limitations associated with the use of *in vitro* systems for studying biofilm infections [133]. For instance, characteristics of the biofilm microenvironment (e.g., nutritional cues, presence of immune cells) [134,135,136] have been shown to influence biofilm morphology and so differences in specific biofilm-forming conditions may limit model applicability.

### 5.2. Ex Vivo Models

*Ex vivo* studies make use of whole, excised corneas that are maintained in an artificial environment before experimentation. Animal corneas are often used due to the limited availability of human corneas and so interspecies variation is one of the main problems with *ex vivo* studies. A lack of standardised methods and paucity of information on animal models means comparing *ex vivo* studies is difficult, and there is dispute regarding the suitability of different animal models. *Ex vivo* models used to investigate bacterial keratitis include mice [137,138,139,140,141], rabbits [140,141,142,143,144,145,146], goats [147], cows [148] and pigs [149,150,151]. It is currently unknown if interspecies differences in the thickness of the corneal epithelium [108,152] and stroma [153,154,155] play a major role in development and progression of infection in the *ex vivo* cornea. Morphological aspects that may affect the development of infection between species have been discussed previously [156] but many questions remain unanswered. Of particular importance is the presence or absence of the Bowman’s layer. The Bowman’s layer is typically found in primate species but has not been found in all animals [157,158] and there is evidence that it functions as an additional barrier to bacterial traversal [159]. The importance of this layer is influenced by the method of infection. Popular infection methods include corneal scarification or intrastromal injection, which bypass the Bowman’s layer and provide direct access to the corneal stroma. In these instances, the protective role of the Bowman’s layer is less important, but other studies have used contact lenses or blotting paper to introduce bacteria without prior wounding of the cornea. Such methods are important for studying intrinsic corneal resistance and/or initial bacterial adhesion, and in these studies, interspecies differences in the Bowman’s layer may compromise model suitability. There are conflicting reports for rabbit and porcine corneas with some studies claiming the Bowman’s layer is absent [160,161,162,163], while others report it as present [164,165]. Given the popularity of these two animal models, it is important that resolution be reached on this topic.

#### Existing *Ex Vivo* Infection Models

**Figure 3:**
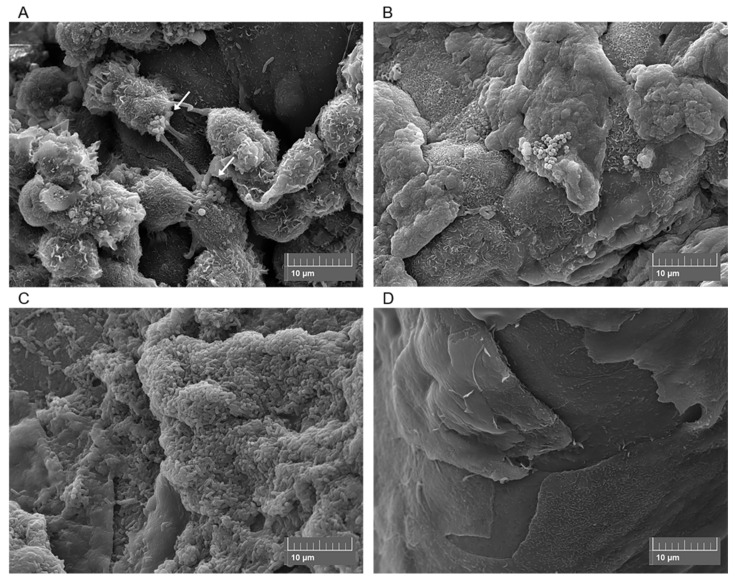
Scanning electron micrographs of *ex vivo* porcine corneas after 4 h Methicillin-Resistant *Staphylococcus aureus* (MRSA) infection (**A**), 6 h MRSA infection (**B**), 24 h *Pseudomonas aeruginosa* infection (**C**) and the uninfected porcine cornea (**D**). Arrows show MRSA adhering to corneal epithelial cells.

Various techniques have been used to induce bacterial infection in *ex vivo* corneas, including prolonged exposure to bacteria [166,167], use of infected contact lenses [149], superficial injury (e.g., tissue paper blotting) [168], corneal scarification [146,147,151] and intrastromal injection [143]. Differences in infection method, inoculum size, culturing techniques, incubation times and bacterial strains mean that comparing *ex vivo* studies is challenging. For example, Pinnock et al. [143] found that more bacteria are recovered after injecting the inoculum into the stroma than after corneal wounding. In contrast, similar infection outcomes were reported for both rabbit and human corneas. Colony Forming Units (CFU) were measured following 24 or 48 h infection and variations in CFU were small despite differences in bacteria and handling techniques for each model [143]. In agreement with Pinnock et al., we recently demonstrated that there was no significant difference in viable cell count between *ex vivo* porcine and rabbit cornea models after 24 h infection, nor when two different strains of *P. aeruginosa* were used [151]. Furthermore, while some studies have reported that infection in *ex vivo* corneas is easy to establish and that progress is visible within less than 24 h [143,151,167], Madhu et al. found that incubation time could be extended by a few days if a smaller inoculum was used [147]. Despite issues with standardisation, *ex vivo* models have been used to study various aspects of bacterial keratitis. This includes: epithelial barrier function [137,169], effect of bacteria on epithelial cell migration [150], bacterial transmission from contact lenses [145,149,170], bacterial adherence to corneal epithelium [168], movement of bacteria in stroma [146], role of virulence factors [139,147] and drug testing of new ophthalmic antimicrobials [132,148]. Despite the popularity of *ex vivo* corneal infection models, biofilm formation under these conditions remains to be characterised. However, our group is currently using an *ex vivo* porcine infection model to study bacterial distribution and biofilm formation at the corneal surface [151] (Figure 3). Scanning Electron Microscopy (SEM) depicts bacterial colonization under different infection conditions, indicating that *ex vivo* porcine models could be useful in the study of established bacterial keratitis infections.

### 5.3. In Vivo Models

*In vivo* modelling involves the use of live animals. Rat [171] and rabbit [172,173,174] models have been reported, but mouse models currently dominate the literature [131,137,138,175,176,177,178]. Despite its smaller size, the murine cornea contains more corneal epithelial cell layers than the human cornea and the ratio of epithelial to stromal cells is larger [179]. As with other animal models, there is a dispute regarding the presence of a Bowman’s layer [164,165] and there are large interspecies differences in immune response that must be considered [180]. However, murine models remain a popular choice for *in vivo* work because of their small size, ease of breeding and the existence of large genetic mutant libraries. Various techniques have been developed for studying bacterial keratitis *in vivo*. Animals are first anesthetized so that corneal wounding/bacterial inoculation can be performed, and infection progresses in the living model. Following scarification, the eyes are enucleated and analysed *ex vivo* or alternatively, intravital imaging techniques have now been reported which allow microscopic analysis to be conducted *in vivo* [181]. *In vivo* corneal models are ideal for studies of host immune defences, inflammation and corneal healing processes. However, these models are not suitable for studying the early stages of infection, as the healthy, intact cornea is difficult to infect unless contaminated contact lenses are used [138,182,183]. Additionally, initiating and developing infection takes days and is not always guaranteed [182].

#### Existing *In Vivo* Infection Models

Increasing interest in ocular biofilms over the past decade has resulted in the development of an established *in vivo* cornea model [177], followed by improved methods of imaging bacteria and biofilm formation [22,131,137,139,176,181]. This has allowed researchers to begin to characterise the process of biofilm formation at the ocular surface (Table 2). *In vivo* infection models have also played an integral role in other areas of bacterial keratitis research, including: biofilm formation on contact lenses in rabbit [172] and mice [175], host–pathogen interactions on ocular samples using proteomics [184,185], activation of immune signalling pathways [186], the role of virulence factors in keratitis [142,173,178,187] and drug testing of new ophthalmic antimicrobials [174,176,188]. Drug testing has included synthetic analogues of host antimicrobial peptides, with one study reporting reduced corneal bioburden and improved ocular scores following treatment with their lead peptide [188]. This suggests that synthetic AMP analogues could provide valuable alternatives/adjuncts to antibiotics and highlights the importance of ocular surface proteins in defence against bacterial keratitis [137,169,189]. For instance, surfactant protein D (SP-D) present in tear fluid has been shown to take part in clearing *P. aeruginosa* from the murine ocular surface [182], while exogenous vasoactive intestinal peptide regulates expression of other proteins involved in infection [190]. However, it was recently found that there are differences in protein expression between human and mouse stroma in vascularized and healthy corneas [191]. These differences are likely to affect pathophysiology between species and may limit the clinical relevance of murine *in vivo* models.

## 6. Conclusions

Bacterial keratitis is a serious corneal infection, characterised by biofilm formation at the ocular surface. Biofilms are notoriously difficult to eradicate because of their increased resistance phenotype. This includes an increased resistance to antimicrobials and biofilm-specific immune evasion mechanisms. To study bacterial keratitis in a meaningful way, it is important that suitable test models exist. The development of *in vitro*, *ex vivo* and *in vivo* models have all made significant contributions to our understanding of bacterial keratitis. Infection models have allowed us to study the role of specific bacterial virulence factors and constituents of the host immune response, as well as the complex interactions that occur between them. However, there are various limitations associated with existing infection models and these limitations must be carefully considered during experimental design and interpretation of results. One particular challenge in the field is the development of co-culture models that display mature biofilm architecture without compromising host viability. As a result, the characterisation of corneal biofilms has mainly been explored *in vivo*. It is important that we continue to optimise corneal biofilm models, as they represent a valuable tool for ophthalmological drug testing. The development of representative models will allow novel therapeutics to be identified more easily and could ultimately help to reduce corneal blindness.

## Figures and Tables

**Table 1:** Evaluation of *in vitro*, *ex vivo* and *in vivo* corneal models for the study of bacterial keratitis infections.

	Advantages	Disadvantages
***in vitro*** **cell culture models**	■ Economical. ■ Reduced use of animals. ■ Cell lines can be used continuously. ■ 3D organotypic models can be developed using multiple cell lines. ■ Many host defence mechanisms remain investigable, e.g. expression of mucins, AMPs, pro-inflammatory cytokines and microRNAs, investigation of cell surface receptors and PRR signalling pathways.	■ Problems with cell lines and genetic drift. ■ Primary cells reach senescence after a few passages. ■ Reduced cell viability and increased susceptibility to infection. ■ Absence of resident and infiltrative immune cells. ■ Absence of conjunctiva. ■ Absence of tear fluid and lacrimal glands. ■ Infection normally occurs under static conditions. ■ Differences in the biofilm microenvironment (e.g. nutritional cues, absence of immune cells) may affect biofilm morphology.
***ex vivo*** **models**	■ Whole-tissue model. ■ Complex 3D surface topology of the cornea is preserved. ■ Increased cell viability facilitates longer infection periods. ■ Presence of resident immune cells.	■ Low availability of human corneas means animal models are commonly used. ■ Lack of standardised infection methods. ■ Dispute regarding corneal anatomy of animal models. ■ Interspecies differences in corneal anatomy, functional characteristics and immune response may affect applicability to human infections. ■ Absence of infiltrative immune cells. ■ Absence of conjunctiva. ■ Absence of tear fluid and lacrimal glands. ■ Infection normally occurs under static conditions. ■ Differences in the biofilm microenvironment (e.g. nutritional cues, absence of immune cells) may affect biofilm morphology.
***in vivo*** **models**	■ Complete immune response (resident/infiltrative immune cells, tear film, conjunctiva and lymphatic vessels). ■ Infection occurs under dynamic, shear stress conditions. ■ Biofilm morphology should be highly similar to the true infectious scenario.	■ Animal models must be used, raising ethical issues. ■ Interspecies differences in corneal anatomy, functional characteristics and immune response may affect applicability to human infections. ■ Expensive. ■ Time-consuming. ■ Infections can be difficult to establish and prior wounding of the cornea is often required.

**Table 2:** Biofilm characteristics of *in vivo* corneal infection models.

Animal model	Pathogen	Biofilm Characteristics	**Ref.**
C57BL/6 black mice	*Pseudomonas aeruginosa* ATCC 9027	■ Rapid shift from planktonic to biofilm lifestyle observed for all corneas. ■ Microcolonies present on day 2 post-infection and fibrous extracellular substances visible. ■ Mature biofilm structures present on day 3. Bacteria form as “mushroom shaped bodies” and “tower like structures” and are embedded in a web of extracellular polysaccharides. ■ A thick, dense biofilm layer is observed on days 5-6. Bacteria become static within this structure. ■ Neutrophils migrate into the corneal stroma and production of NETs is observed at early time points. Neutrophils are localised to the biofilm surface once mature biofilm structures develop.	[22]
C57BL/6 and Swiss Webster (SW) mice	*Pseudomonas aeruginosa* PAO1-GFP and 6294-GFP (clinical isolate)	■ Early (12 h) biofilms are composed of bacterial clusters/microcolonies that are thought to emanate from the infected epithelial cells. ■ Late (24 h) biofilms are composed of bacterial sheets. ■ Biofilm bacteria are surrounded by Psl polysaccharide but there is a low abundance of alginate. ■ Biofilms are resistant to neutrophil infiltration.	[176]
BALB/c mice	*Staphylococcus aureus* and *Fusarium falciforme* (clinical isolates)	■ A mixed biofilm is observed after 72 h. ■ *S. aureus*: Bacteria colonise the corneal epithelium and a part of the stroma. Bacterial clusters observed, including a large cocci aggregate at the site of the corneal lesion. Bacteria secrete exopolysaccharides that form “halos” around the bacteria and then merge with the extracellular matrix of other cocci. Development of a new blood vessel in the stroma is observed and attributed to the host immune response. ■ *F. falciforme*: Hyphae and conidia observed and hyphae migrates through stroma to reach the endothelium. *F. falciforme* structures are embedded in a fibrin matrix within the stroma. Presence/growth of fungi causes corneal collagen fibres to become disorganised.	[131]

## References

[1] Keay L., Edwards K., Naduvilath T., Taylor H.R., Snibson G.R., Forde K., Stapleton F. (2006). Microbial keratitis—Predisposing factors and morbidity. Ophthalmology.

[2] Keay L., Edwards K., Stapleton F. (2009). Signs, Symptoms, and Comorbidities in Contact Lens-Related Microbial Keratitis. Optom. Vis. Sci..

[3] Pascolini D., Mariotti S.P. (2012). Global estimates of visual impairment: 2010. Br. J. Ophthalmol..

[4] Whitcher J.P., Srinivasan M., Upadhyay M.P. (2001). Corneal blindness: A global perspective. Bull. World Health Organ..

[5] Collier S.A., Gronostaj M.P., MacGurn A.K., Cope J.R., Awsumb K.L., Yoder J.S., Beach M.J. (2014). Estimated Burden of Keratitis—United States, 2010. Mmwr-Morb. Mortal. Wkly. Rep..

[6] Houang E., Lam D., Fan D., Seal D. (2001). Microbial keratitis in Hong Kong: Relationship to climate, environment and contact-lens disinfection. Trans. R. Soc. Trop. Med. Hyg..

[7] Neumann M., Sjostrand J. (1993). Central Microbial Keratitis in a Swedish City Population—A 3-Year Prospective-Study in Gothenburg. Acta Ophthalmol..

[8] Alexandrakis G., Alfonso E.C., Miller D. (2000). Shifting trends in bacterial keratitis in South Florida and emerging resistance to fluoroquinolones. Ophthalmology.

[9] Fleiszig S.M.J., Kroken A.R., Nieto V., Grosser M.R., Wan S.J., Metruccio M.M.E., Evans D.J. (2019). Contact lens-related corneal infection: Intrinsic resistance and its compromise. Prog. Retin. Eye Res..

[10] Bourcier T., Thomas F., Borderie V., Chaumeil C., Laroche L. (2003). Bacterial keratitis: Predisposing factors, clinical and microbiological review of 300 cases. Br. J. Ophthalmol..

[11] Ng A.L.K., To K.K.W., Choi C.C.L., Yuen L.H., Yim S.M., Chan K.S.K., Lai J.S.M., Wong I.Y.H. (2015). Predisposing Factors, Microbial Characteristics, and Clinical Outcome of Microbial Keratitis in a Tertiary Centre in Hong Kong: A 10-Year Experience. J. Ophthalmol..

[12] Ung L., Bispo P.J.M., Shanbhag S.S., Gilmore M.S., Chodosh J. (2019). The persistent dilemma of microbial keratitis: Global burden, diagnosis, and antimicrobial resistance. Surv. Ophthalmol..

[13] Bharathi M.J., Ramakrishnan R., Meenakshi R., Padmavathy S., Shivakumar C., Srinivasan M. (2007). Microbial keratitis in South India: Influence of risk factors, climate, and geographical variation. Ophthalmic Epidemiol..

[14] Al-Mujaini A., Al-Kharusi N., Thakral A., Wali U.K. (2009). Bacterial keratitis: Perspective on epidemiology, clinico-pathogenesis, diagnosis and treatment. Sultan Qaboos Univ. Med. J..

[15] Chidambaram J.D., Prajna N.V., Srikanthi P., Lanjewar S., Shah M., Elakkiya S., Lalitha P., Burton M.J. (2018). Epidemiology, risk factors, and clinical outcomes in severe microbial keratitis in South India. Ophthalmic Epidemiol..

[16] Sagerfors S., Ejdervik-Lindblad B., Soderquist B. (2019). Infectious keratitis: Isolated microbes and their antibiotic susceptibility pattern during 2004-2014 in Region Orebro County, Sweden. Acta Ophthalmol..

[17] Erie J.C., Nevitt M.P., Hodge D.O., Ballard D. (1993). Incidence of ulcerative keratitis in a defined population from 1950 through 1988. Arch. Ophthalmol..

[18] Donlan R.M., Costerton J.W. (2002). Biofilms: Survival mechanisms of clinically relevant microorganisms. Clin. Microbiol. Rev..

[19] Veerachamy S., Yarlagadda T., Manivasagam G., Yarlagadda P. (2014). Bacterial adherence and biofilm formation on medical implants: A review. Proc. Inst. Mech. Eng. Part H-J. Eng. Med..

[20] Zegans M.E., Shanks R.M.Q., Toole G.A. (2005). Bacterial biofilms and ocular infections. Ocul. Surf..

[21] Bispo P.J.M., Haas W., Gilmore M.S. (2015). Biofilms in Infections of the Eye. Pathogens.

[22] Saraswathi P., Beuerman R.W. (2015). Corneal Biofilms: From Planktonic to Microcolony Formation in an Experimental Keratitis Infection with *Pseudomonas aeruginosa*. Ocul. Surf..

[23] Zegans M.E., DiGiandomenico A., Ray K., Naimie A., Keller A.E., Stover C.K., Lalitha P., Srinivasan M., Acharya N.R., Lietman T.M. (2016). Association of Biofilm Formation, Psl Exopolysaccharide Expression, and Clinical Outcomes in *Pseudomonas aeruginosa* Keratitis Analysis of Isolates in the Steroids for Corneal Ulcers Trial. Jama Ophthalmol..

[24] Dave A., Samarth A., Karolia R., Sharma S., Karunakaran E., Partridge L., MacNeil S., Monk P.N., Garg P., Roy S. (2020). Characterization of Ocular Clinical Isolates of *Pseudomonas aeruginosa* from Non-Contact Lens Related Keratitis Patients from South India. Microorganisms.

[25] O’Toole G., Kaplan H.B., Kolter R. (2000). Biofilm formation as microbial development. Annu. Rev. Microbiol..

[26] Hoiby N., Ciofu O., Johansen H.K., Song Z.J., Moser C., Jensen P.O., Molin S., Givskov M., Tolker-Nielsen T., Bjarnsholt T. (2011). The clinical impact of bacterial biofilms. Int. J. Oral Sci..

[27] Stewart P., Costerton J.W. (2001). Antibiotic Resistance of Bacteria in Biofilms. Lancet.

[28] Hanke M.L., Kielian T. (2012). Deciphering mechanisms of staphylococcal biofilm evasion of host immunity. Front. Cell. Infect. Microbiol..

[29] Rybtke M., Hultqvist L.D., Givskov M., Tolker-Nielsen T. (2015). *Pseudomonas aeruginosa* Biofilm Infections: Community Structure, Antimicrobial Tolerance and Immune Response. J. Mol. Biol..

[30] Buhmann M.T., Stiefel P., Maniura-Weber K., Ren Q. (2016). *In Vitro* Biofilm Models for Device-Related Infections. Trends Biotechnol..

[31] Cho P., Boost M.V. (2019). Evaluation of prevention and disruption of biofilm in contact lens cases. Ophthalmic Physiol. Opt..

[32] Kackar S., Suman E., Kotian M.S. (2017). Bacterial and Fungal Biofilm formation on Contact Lenses and their Susceptibility to Lens Care Solutions. Indian J. Med. Microbiol..

[33] Henriques M., Sousa C., Lira M., Elisabete M., Oliveira R., Azeredo J. (2005). Adhesion of *Pseudomonas aeruginosa* and Staphylococcus epidermidis to silicone-hydrogel contact lenses. Optom. Vis. Sci..

[34] Dutta D., Cole N., Willcox M. (2012). Factors influencing bacterial adhesion to contact lenses. Mol. Vis..

[35] Hsiao Y.-T., Fang P.-C., Chen J.-L., Hsu S.-L., Chao T.-L., Yu H.-J., Lai Y.-H., Huang Y.-T., Kuo M.-T. (2018). Molecular Bioburden of the Lens Storage Case for Contact Lens-Related Keratitis. Cornea.

[36] Pendleton J.N., Gorman S.P., Gilmore B.F. (2013). Clinical relevance of the ESKAPE pathogens. Expert Rev. Anti-Infect. Ther..

[37] Santajit S., Indrawattana N. (2016). Mechanisms of Antimicrobial Resistance in ESKAPE Pathogens. Biomed Res. Int..

[38] World Health Organization WHO Publishes List Of Bacteria for Which New Antibiotics Are Urgently Needed. https://www.who.int/news-room/detail/27-02-2017-who-publishes-list-of-bacteria-for-which-new-antibiotics-are-urgently-needed.

[39] Tam A.L.C., Cote E., Saldanha M., Lichtinger A., Slomovic A.R. (2017). Bacterial Keratitis in Toronto: A 16-Year Review of the Microorganisms Isolated and the Resistance Patterns Observed. Cornea.

[40] Chang V.S., Dhaliwal D.K., Raju L., Kowalski R.P. (2015). Antibiotic Resistance in the Treatment of Staphylococcus aureus Keratitis: A 20-Year Review. Cornea.

[41] Tuft S., Burton M. The Royal College Ophthalmologists, Focus: Microbial Keratitis. https://www.rcophth.ac.uk/wp-content/uploads/2014/08/Focus-Autumn-2013.pdf.

[42] Saffari M., Karami S., Firoozeh F., Sehat M. (2017). Evaluation of biofilm-specific antimicrobial resistance genes in *Pseudomonas aeruginosa* isolates in Farabi Hospital. J. Med. Microbiol..

[43] Heidari H., Hadadi M., Ebrahim-Saraie H.S., Mirzaei A., Taji A., Hosseini S.R., Motamedifar M. (2018). Characterization of virulence factors, antimicrobial resistance patterns and biofilm formation of *Pseudomonas aeruginosa* and Staphylococcus spp. strains isolated from corneal infection. J. Fr. D’ophtalmol..

[44] Centers for Disease Control and Prevention (2013). Antibiotic resistance threats in the United States, 2013.

[45] Kaye S., Tuft S., Neal T., Tole D., Leeming J., Figueiredo F., Armstrong M., McDonnell P., Tullo A., Parry C. (2010). Bacterial Susceptibility to Topical Antimicrobials and Clinical Outcome in Bacterial Keratitis. Investig. Ophthalmol. Vis. Sci..

[46] Walsh C. (2000). Molecular mechanisms that confer antibacterial drug resistance. Nature.

[47] Anwar H., Vanbiesen T., Dasgupta M., Lam K., Costerton J.W. (1989). Interaction of biofilm bacteria with antibiotics in a novel invitro chemostat system. Antimicrob. Agents Chemother..

[48] Hall C.W., Mah T.F. (2017). Molecular mechanisms of biofilm-based antibiotic resistance and tolerance in pathogenic bacteria. FEMS Microbiol. Rev..

[49] Olsen I. (2015). Biofilm-specific antibiotic tolerance and resistance. Eur. J. Clin. Microbiol. Infect. Dis..

[50] Stewart P.S. (1996). Theoretical aspects of antibiotic diffusion into microbial biofilms. Antimicrob. Agents Chemother..

[51] Mah T.F., Pitts B., Pellock B., Walker G.C., Stewart P.S., O’Toole G.A. (2003). A genetic basis for *Pseudomonas aeruginosa* biofilm antibiotic resistance. Nature.

[52] Hentzer M., Teitzel G.M., Balzer G.J., Heydorn A., Molin S., Givskov M., Parsek M.R. (2001). Alginate overproduction affects *Pseudomonas aeruginosa* biofilm structure and function. J. Bacteriol..

[53] Albayaty Y.N., Thomas N., Hasan S., Prestidge C.A. (2018). Penetration of topically used antimicrobials through Staphylococcus aureus biofilms: A comparative study using different models. J. Drug Deliv. Sci. Technol..

[54] Darouiche R.O., Dhir A., Miller A.J., Landon G.C., Raad I.I., Musher D.M. (1994). Vancomycin penetration into biofilm covering infected prostheses and effect on bacteria. J. Infect. Dis..

[55] Sepandj F., Ceri H., Gibb A., Read R., Olson M. (2004). Minimum inhibitory concentration (MIC) versus minimum biofilm eliminating concentration (MBEC) in evaluation of antibiotic sensitivity of gram-negative bacilli causing peritonitis. Perit. Dial. Int..

[56] Walters M.C., Roe F., Bugnicourt A., Franklin M.J., Stewart P.S. (2003). Contributions of antibiotic penetration, oxygen limitation, and low metabolic activity to tolerance of *Pseudomonas aeruginosa* biofilms to ciprofloxacin and tobramycin. Antimicrob. Agents Chemother..

[57] Drenkard E. (2003). Antimicrobial resistance of *Pseudomonas aeruginosa* biofilms. Microbes Infect..

[58] Tack K.J., Sabath L.D. (1985). Increased minimum inhibitory concentrations with anaerobiasis for tobramycin, gentamicin, and amikacin, compared to latamoxef, piperacillin, chloramphenicol, and clindamycin. Chemotherapy.

[59] Bigger J.W. (1944). Treatment of staphylococcal infections with penicillin—By intermittent sterilisation. Lancet.

[60] Fisher R.A., Gollan B., Helaine S. (2017). Persistent bacterial infections and persister cells. Nat. Rev. Microbiol..

[61] Lewis K. (2005). Persister cells and the riddle of biofilm survival. Biochemistry.

[62] Balaban N.Q., Merrin J., Chait R., Kowalik L., Leibler S. (2004). Bacterial persistence as a phenotypic switch. Science.

[63] Askarian F., Wagner T., Johannessen M., Nizet V. (2018). Staphylococcus aureus modulation of innate immune responses through Toll-like (TLR), (NOD)-like (NLR) and C-type lectin (CLR) receptors. FEMS Microbiol. Rev..

[64] Kimbrell D.A., Beutler B. (2001). The evolution and genetics of innate immunity. Nat. Rev. Genet..

[65] Rigby K.M., DeLeo F.R. (2012). Neutrophils in innate host defense against Staphylococcus aureus infections. Semin. Immunopathol..

[66] Olivares E., Badel-Berchoux S., Provot C., Prevost G., Bernardi T., Jehl F. (2020). Clinical Impact of Antibiotics for the Treatment of *Pseudomonas aeruginosa* Biofilm Infections. Front. Microbiol..

[67] Arciola C.R., Campoccia D., Montanaro L. (2018). Implant infections: Adhesion, biofilm formation and immune evasion. Nat. Rev. Microbiol..

[68] Jensen P.O., Givskov M., Bjarnsholt T., Moser C. (2010). The immune system vs. *Pseudomonas aeruginosa* biofilms. FEMS Immunol. Med. Microbiol..

[69] Campoccia D., Mirzaei R., Montanaro L., Arciola C.R. (2019). Hijacking of immune defences by biofilms: A multifront strategy. Biofouling.

[70] de Vor L., Rooijakkers S.H.M., van Strijp J.A.G. (2020). Staphylococci evade the innate immune response by disarming neutrophils and forming biofilms. FEBS Lett..

[71] Thurlow L.R., Hanke M.L., Fritz T., Angle A., Aldrich A., Williams S.H., Engebretsen I.L., Bayles K.W., Horswill A.R., Kielian T. (2011). Staphylococcus aureus Biofilms Prevent Macrophage Phagocytosis and Attenuate Inflammation *in vivo*. J. Immunol..

[72] Scherr T.D., Hanke M.L., Huang O.W., James D.B.A., Horswill A.R., Bayles K.W., Fey P.D., Torres V.J., Kielian T. (2015). Staphylococcus aureus Biofilms Induce Macrophage Dysfunction Through Leukocidin AB and Alpha-Toxin. MBio.

[73] Herant M., Heinrich V., Dembo M. (2006). Mechanics of neutrophil phagocytosis: Experiments and quantitative models. J. Cell Sci..

[74] Kovach K., Davis-Fields M., Irie Y., Jain K., Doorwar S., Vuong K., Dhamani N., Mohanty K., Touhami A., Gordon V.D. (2017). Evolutionary adaptations of biofilms infecting cystic fibrosis lungs promote mechanical toughness by adjusting polysaccharide production. Npj Biofilms Microbiomes.

[75] Zapotoczna M., McCarthy H., Rudkin J.K., O’Gara J.P., O’Neill E. (2015). An Essential Role for Coagulase in Staphylococcus aureus Biofilm Development Reveals New Therapeutic Possibilities for Device-Related Infections. J. Infect. Dis..

[76] Thammavongsa V., Kim H.K., Missiakas D., Schneewind O. (2015). Staphylococcal manipulation of host immune responses. Nat. Rev. Microbiol..

[77] Kristian S.A., Birkenstock T.A., Sauder U., Mack D., Gotz F., Landmann R. (2008). Biofilm formation induces C3a release and protects Staphylococcus epidermidis from IgG and complement deposition and from neutrophil-dependent killing. J. Infect. Dis..

[78] Cerca N., Jefferson K.K., Oliveira R., Pier G.B., Azeredo J. (2006). Comparative antibody-mediated phagocytosis of Staphylococcus epidermidis cells grown in a biofilm or in the planktonic state. Infect. Immun..

[79] Leid J.G., Willson C.J., Shirtliff M.E., Hassett D.J., Parsek M.R., Jeffers A.K. (2005). The exopolysaccharide alginate protects *Pseudomonas aeruginosa* biofilm bacteria from IFN-gamma-mediated macrophage killing. J. Immunol..

[80] Pier G.B., Coleman F., Grout M., Franklin M., Ohman D.E. (2001). Role of alginate O acetylation in resistance of mucoid *Pseudomonas aeruginosa* to opsonic phagocytosis. Infect. Immun..

[81] Mishra M., Byrd M.S., Sergeant S., Azad A.K., Parsek M.R., McPhail L., Schlesinger L.S., Wozniak D.J. (2012). *Pseudomonas aeruginosa* Psl polysaccharide reduces neutrophil phagocytosis and the oxidative response by limiting complement-mediated opsonization. Cell. Microbiol..

[82] Novick R.P. (2003). Autoinduction and signal transduction in the regulation of staphylococcal virulence. Mol. Microbiol..

[83] He L., Le K.Y., Khan B.A., Nguyen T.H., Hunt R.L., Bae J.S., Kabat J., Zheng Y., Cheung G.Y.C., Li M. (2019). Resistance to leukocytes ties benefits of quorum sensing dysfunctionality to biofilm infection. Nat. Microbiol..

[84] Periasamy S., Joo H.S., Duong A.C., Bach T.H.L., Tan V.Y., Chatterjee S.S., Cheung G.Y.C., Otto M. (2012). How Staphylococcus aureus biofilms develop their characteristic structure. Proc. Natl. Acad. Sci. USA.

[85] Vuong C., Saenz H.L., Gotz F., Otto M. (2000). Impact of the agr quorum-sensing system on adherence to polystyrene in Staphylococcus aureus. J. Infect. Dis..

[86] Collins J., Rudkin J., Recker M., Pozzi C., O’Gara J.P., Massey R.C. (2010). Offsetting virulence and antibiotic resistance costs by MRSA. ISME J..

[87] Painter K.L., Krishna A., Wigneshweraraj S., Edwards A.M. (2014). What role does the quorum-sensing accessory gene regulator system play during Staphylococcus aureus bacteremia?. Trends Microbiol..

[88] Valentini M., Filloux A. (2016). Biofilms and Cyclic di-GMP (c-di-GMP) Signaling: Lessons from *Pseudomonas aeruginosa* and Other Bacteria. J. Biol. Chem..

[89] Luo Y., Zhao K., Baker A.E., Kuchma S.L., Coggan K.A., Wolfgang M.C., Wong G.C.L., O’Toole G.A. (2015). A Hierarchical Cascade of Second Messengers Regulates *Pseudomonas aeruginosa* Surface Behaviors. MBio.

[90] Riquelme S.A., Ahn D., Prince A. (2018). *Pseudomonas aeruginosa* and Klebsiella pneumoniae Adaptation to Innate Immune Clearance Mechanisms in the Lung. J. Innate Immun..

[91] Cohen T.S., Prince A.S. (2013). Activation of inflammasome signaling mediates pathology of acute P. aeruginosa pneumonia. J. Clin. Investig..

[92] Huus K.E., Joseph J., Zhang L., Wong A., Aaron S.D., Mah T.F., Sad S. (2016). Clinical Isolates of *Pseudomonas aeruginosa* from Chronically Infected Cystic Fibrosis Patients Fail To Activate the Inflammasome during Both Stable Infection and Pulmonary Exacerbation. J. Immunol..

[93] Jensen E.T., Kharazmi A., Hoiby N., Costerton J.W. (1992). Some bacterial parameters influencing the neutrophil oxidative burst response to pseudomonas-aeruginosa biofilms. Apmis.

[94] Guenther F., Stroh P., Wagner C., Obst U., Hansch G.M. (2009). Phagocytosis of staphylococci biofilms by polymorphonuclear neutrophils: S. aureus and S. epidermidis differ with regard to their susceptibility towards the host defense. Int. J. Artif. Organs.

[95] Tateda K., Ishii Y., Horikawa M., Matsumoto T., Miyairi S., Pechere J.C., Standiford T.J., Ishiguro M., Yamaguchi K. (2003). The *Pseudomonas aeruginosa* autoinducer N-3-oxododecanoyl homoserine lactone accelerates apoptosis in macrophages and neutrophils. Infect. Immun..

[96] Jensen P.O., Bjarnsholt T., Phipps R., Rasmussen T.B., Calum H., Christoffersen L., Moser C., Williams P., Pressler T., Givskov M. (2007). Rapid necrotic killing of polymorphonuclear leukocytes is caused by quorum-sensing-controlled production of rhamnolipid by *Pseudomonas aeruginosa*. Microbiology.

[97] Buchan K.D., Foster S.J., Renshaw S.A. (2019). Staphylococcus aureus: Setting its sights on the human innate immune system. Microbiology.

[98] Parks Q.M., Young R.L., Poch K.R., Malcolm K.C., Vasil M.L., Nick J.A. (2009). Neutrophil enhancement of *Pseudomonas aeruginosa* biofilm development: Human F-actin and DNA as targets for therapy. J. Med. Microbiol..

[99] Lewenza S. (2013). Extracellular DNA-induced antimicrobial peptide resistance mechanisms in *Pseudomonas aeruginosa*. Front. Microbiol..

[100] Robertson D.M., Parks Q.M., Young R.L., Kret J., Poch K.R., Malcolm K.C., Nichols D.P., Nichols M., Zhu M.F., Cavanagh H.D. (2011). Disruption of Contact Lens-Associated *Pseudomonas aeruginosa* Biofilms Formed in the Presence of Neutrophils. Investig. Ophthalmol. Vis. Sci..

[101] Brinkmann V., Reichard U., Goosmann C., Fauler B., Uhlemann Y., Weiss D.S., Weinrauch Y., Zychlinsky A. (2004). Neutrophil extracellular traps kill bacteria. Science.

[102] Thanabalasuriar A., Scott B.N.V., Peiseler M., Willson M.E., Zeng Z.T., Warrener P., Keller A.E., Surewaard B.G.J., Dozier E.A., Korhonen J.T. (2019). Neutrophil Extracellular Traps Confine *Pseudomonas aeruginosa* Ocular Biofilms and Restrict Brain Invasion. Cell Host Microbe.

[103] Berends E.T.M., Horswill A.R., Haste N.M., Monestier M., Nizet V., von Kockritz-Blickwede M. (2010). Nuclease Expression by Staphylococcus aureus Facilitates Escape from Neutrophil Extracellular Traps. J. Innate Immun..

[104] Winstel V., Schneewind O., Missiakas D. (2019). Staphylococcus aureus Exploits the Host Apoptotic Pathway To Persist during Infection. MBio.

[105] Thammavongsa V., Missiakas D.M., Schneewind O. (2013). Staphylococcus aureus Degrades Neutrophil Extracellular Traps to Promote Immune Cell Death. Science.

[106] Gries C.M., Bruger E.L., Moormeier D.E., Scherr T.D., Waters C.M., Kielian T. (2016). Cyclic di-AMP Released from Staphylococcus aureus Biofilm Induces a Macrophage Type I Interferon Response. Infect. Immun..

[107] Gonzalez J.F., Hahn M.M., Gunn J.S. (2018). Chronic biofilm-based infections: Skewing of the immune response. Pathog. Dis..

[108] Krachmer J.H., Mannis M.J., Holland E.J. (2010). Cornea and Sclera: Anatomy and Physiology. Cornea.

[109] Dua H.S., Faraj L.A., Said D.G., Gray T., Lowe J. (2013). Human Corneal Anatomy Redefined A Novel Pre-Descemet’s Layer (Dua’s Layer). Ophthalmology.

[110] Sosnova-Netukova M., Kuchynka P., Forrester J.V. (2007). The suprabasal layer of corneal epithelial cells represents the major barrier site to the passive movement of small molecules and trafficking leukocytes. Br. J. Ophthalmol..

[111] Ranta V.P., Laavola M., Toropainen E., Vellonen K.S., Talvitie A., Urtti A. (2003). Ocular pharmacokinetic modeling using corneal absorption and desorption rates from *in vitro* permeation experiments with cultured corneal epithelial cells. Pharm. Res..

[112] Toropainen E. (2007). Corneal Epithelial Cell Culture Model for Pharmaceutical Studies. Ph.D. Dissertation.

[113] Kaluzhny Y., Kinuthia M.W., Truong T., Lapointe A.M., Hayden P., Klausner M. (2018). New Human Organotypic Corneal Tissue Model for Ophthalmic Drug Delivery Studies. Investig. Ophthalmol. Vis. Sci..

[114] Zorn-Kruppa M., Tykhonova S., Belge G., Bednarz J., Diehl H.A., Engelke M. (2005). A human corneal equivalent constructed from SV40-immortalised corneal cell lines. Atla-Altern. Lab. Anim..

[115] Builles N., Bechetoille N., Justin V., Andre V., Barbaro V., Di Iorio E., Auxenfans C., Hulmes D.J.S., Damour O. (2007). Development of a hemicornea from human primary cell cultures for pharmacotoxicology testing. Cell Biol. Toxicol..

[116] Kahn C.R., Young E., Lee I.H., Rhim J.S. (1993). Human corneal epithelial primary cultures and cell-lines with extended life-span-in-vitro model for ocular studies. Investig. Ophthalmol. Vis. Sci..

[117] Ouellette M.M., McDaniel L.D., Wright W.E., Shay J.W., Schultz R.A. (2000). The establishment of telomerase-immortalized cell lines representing human chromosome instability syndromes. Hum. Mol. Genet..

[118] Hughes P., Marshall D., Reid Y., Parkes H., Gelber C. (2007). The costs of using unauthenticated, over-passaged cell lines: How much more data do we need?. Biotechniques.

[119] Greco D., Vellonen K.S., Turner H.C., Hakli M., Tervo T., Auvinen P., Wolosin J.M., Urtti A. (2010). Gene expression analysis in SV-40 immortalized human corneal epithelial cells cultured with an air-liquid interface. Mol. Vis..

[120] Postnikoff C.K., Pintwala R., Williams S., Wright A.M., Hileeto D., Gorbet M.B. (2014). Development of a Curved, Stratified, *In Vitro* Model to Assess Ocular Biocompatibility. PLoS ONE.

[121] Toropainen E., Ranta V.P., Talvitie A., Suhonen P., Urtti A. (2001). Culture model of human corneal epithelium for prediction of ocular drug absorption. Investig. Ophthalmol. Vis. Sci..

[122] Toropainen E., Ranta V.P., Vellonen K.S., Palmgren J., Talvitie A., Laavola M., Suhonen P., Hamalainen K.M., Auriola S., Urtti A. (2003). Paracellular and passive transcellular permeability in immortalized human corneal epithelial cell culture model. Eur. J. Pharm. Sci..

[123] Reichl S., Kolln C., Hahne M., Verstraelen J. (2011). *In vitro* cell culture models to study the corneal drug absorption. Expert Opin. Drug Metab. Toxicol..

[124] Dey S. (2011). Corneal cell culture models: A tool to study corneal drug absorption. Expert Opin. Drug Metab. Toxicol..

[125] Jett B.D., Gilmore M.S. (2002). Internalization of Staphylococcus aureus by human corneal epithelial cells: Role of bacterial fibronectin-binding protein and host cell factors. Infect. Immun..

[126] Garcia B., Merayo-Lloves J., Rodriguez D., Alcalde I., Garcia-Suarez O., Alfonso J.F., Baamonde B., Fernandez-Vega A., Vazquez F., Quiros L.M. (2016). Different Use of Cell Surface Glycosaminoglycans As Adherence Receptors to Corneal Cells by Gram Positive and Gram Negative Pathogens. Front. Cell. Infect. Microbiol..

[127] Sharma P., Guha S., Garg P., Roy S. (2018). Differential expression of antimicrobial peptides in corneal infection and regulation of antimicrobial peptides and reactive oxygen species by type III secretion system of &IT*Pseudomonas aeruginosa* & IT. Pathog. Dis..

[128] Gipson I.K., Spurr-Michaud S., Tisdale A., Menon B.B. (2014). Comparison of the Transmembrane Mucins MUC1 and MUC16 in Epithelial Barrier Function. PLoS ONE.

[129] Fleiszig S.M.J., Kwong M.S.F., Evans D.J. (2003). Modification of *Pseudomonas aeruginosa* interactions with corneal epithelial cells by human tear fluid. Infect. Immun..

[130] Kwong M.S.F., Evans D.J., Ni M., Cowell B.A., Fleiszig S.M.J. (2007). Human tear fluid protects against *Pseudomonas aeruginosa* keratitis in a murine experimental model. Infect. Immun..

[131] Ponce-Angulo D.G., Bautista-Hernandez L.A., Calvillo-Medina R.P., Castro-Tecorral F.I., Aparicio-Ozores G., Lopez-Villegas E.O., Ribas-Aparicio R.M., Bautista-de Lucio V.M. (2020). Microscopic characterization of biofilm in mixed keratitis in a novel murine model. Microb. Pathog..

[132] Doroshenko N., Rimmer S., Hoskins R., Garg P., Swift T., Spencer H.L.M., Lord R.M., Katsikogianni M., Pownall D., MacNeil S. (2018). Antibiotic functionalised polymers reduce bacterial biofilm and bioburden in a simulated infection of the cornea. Biomater. Sci..

[133] Roberts A.E.L., Kragh K.N., Bjarnsholt T., Diggle S.P. (2015). The Limitations of *In Vitro* Experimentation in Understanding Biofilms and Chronic Infection. J. Mol. Biol..

[134] Palmer K.L., Aye L.A., Whiteley M. (2007). Nutritional cues control *Pseudomonas aeruginosa* multicellular Behavior in cystic fibrosis sputum. J. Bacteriol..

[135] Bjarnsholt T., Alhede M., Eickhardt-Sorensen S.R., Moser C., Kuhl M., Jensen P.O., Hoiby N. (2013). The *in vivo* biofilm. Trends Microbiol..

[136] Kolpen M., Bjarnsholt T., Moser C., Hansen C.R., Rickelt L.F., Kuhl M., Hempel C., Pressler T., Hoiby N., Jensen P.O. (2014). Nitric oxide production by polymorphonuclear leucocytes in infected cystic fibrosis sputum consumes oxygen. Clin. Exp. Immunol..

[137] Metruccio M.M.E., Tam C., Evans D.J., Xie A.L., Stern M.E., Fleiszig S.M.J. (2017). Contributions of MyD88-dependent receptors and CD11c-positive cells to corneal epithelial barrier function against *Pseudomonas aeruginosa*. Sci. Rep..

[138] Metruccio M.M.E., Wan S.J., Horneman H., Kroken A.R., Sullivan A.B., Truong T.N., Mun J.J., Tam C.K.P., Frith R., Welsh L. (2019). A novel murine model for contact lens wear reveals clandestine IL-1R dependent corneal parainflammation and susceptibility to microbial keratitis upon inoculation with *Pseudomonas aeruginosa*. Ocul. Surf..

[139] Sullivan A.B., Tam K.P.C., Metruccio M.M.E., Evans D.J., Fleiszig S.M.J. (2015). The Importance of the *Pseudomonas aeruginosa* Type III Secretion System in Epithelium Traversal Depends upon Conditions of Host Susceptibility. Infect. Immun..

[140] Zhu B., Liu C.L., Liu S.H., Cong H.J., Chen Y.H., Gu L.C., Ma L.Y.Z. (2016). Membrane association of SadC enhances its diguanylate cyclase activity to control exopolysaccharides synthesis and biofilm formation in *Pseudomonas aeruginosa*. Environ. Microbiol..

[141] Zhu H., Kochevar I.E., Behlau I., Zhao J., Wang F.H., Wang Y.C., Sun X.D., Hamblin M.R., Dai T.H. (2017). Antimicrobial Blue Light Therapy for Infectious Keratitis: *Ex Vivo* and *In Vivo* Studies. Investig. Ophthalmol. Vis. Sci..

[142] Hume E.B.H., Dajcs J.J., Moreau J.M., Sloop G.D., Willcox M.D.P., O’Callaghan R.J. (2001). Staphylococcus corneal virulence in a new topical model of infection. Investig. Ophthalmol. Vis. Sci..

[143] Pinnock A., Shivshetty N., Roy S., Rimmer S., Douglas I., MacNeil S., Garg P. (2017). *Ex vivo* rabbit and human corneas as models for bacterial and fungal keratitis. Graefes Arch. Clin. Exp. Ophthalmol..

[144] Lawinbrussel C.A., Refojo M.F., Leong F.L., Hanninen L., Kenyon K.R. (1993). Effect of pseudomonas-aeruginosa concentration in experimental contact lens-related microbial keratitis. Cornea.

[145] Ren H., Petroll W., Jester J., Cavanagh H., Mathers W., Bonnano J., Kennedy R. (1997). Adherence of *Pseudomonas aeruginosa* to shed rabbit corneal epithelial cells after overnight wear of contact lenses. Contact Lens Assoc. Ophthalmol. J..

[146] Robertson D.M., Rogers N.A., Petroll W.M., Zhu M.F. (2017). Second harmonic generation imaging of corneal stroma after infection by *Pseudomonas aeruginosa*. Sci. Rep..

[147] Madhu S.N., Jha K.K., Karthyayani A.P., Gajjar D.U. (2018). *Ex vivo* Caprine Model to Study Virulence Factors in Keratitis. J. Ophthalmic Vis. Res..

[148] Chu H.S., Hu F.R., Chen C.T. (2013). Photodynamic antimicrobial chemotherapy for methicillin-resistant Staphylococcus aureus -*in vitro*, biofilm, and *ex vivo* bovine keratitis model. Investig. Ophthalmol. Vis. Sci..

[149] Vermeltfoort P.B.J., van Kooten T.G., Bruinsma G.M., Hooymans A.M.M., van der Mei H.C., Busscher H.J. (2005). Bacterial transmission from contact lenses to porcine corneas: An *ex vivo* study. Investig. Ophthalmol. Vis. Sci..

[150] Brothers K.M., Stella N.A., Hunt K.M., Romanowski E.G., Liu X.Y., Klarlund J.K., Shanks R.M.Q. (2015). Putting on the brakes: Bacterial impediment of wound healing. Sci. Rep..

[151] Okurowska K., Roy S., Thokala P., Partridge L., Garg P., MacNeil S., Monk P.N., Karunakaran E. (2020). Establishing a Porcine *Ex Vivo* Cornea Model for Studying Drug Treatments against Bacterial Keratitis. J. Vis. Exp..

[152] Agarwal P., Rupenthal I.D. (2016). *In vitro* and *ex vivo* corneal penetration and absorption models. Drug Deliv. Transl. Res..

[153] Hatami-Marbini H., Etebu E., Rahimi A. (2013). Swelling Pressure and Hydration Behavior of Porcine Corneal Stroma. Curr. Eye Res..

[154] Ehlers N., Heegaard S., Hjortdal J., Ivarsen A., Nielsen K., Prause J.U. (2010). Morphological evaluation of normal human corneal epithelium. Acta Ophthalmol..

[155] Abhari S., Eisenback M., Kaplan H.J., Walters E., Prather R.S., Scott P.A. (2018). Anatomic Studies of the Miniature Swine Cornea. Anat. Rec. -Adv. Integr. Anat. Evol. Biol..

[156] Marquart M.E. (2011). Animal Models of Bacterial Keratitis. J. Biomed. Biotechnol..

[157] Merindano M.D., Costa J., Canals M., Potau J.M., Ruano D. (2002). A comparative study of Bowman’s layer in some mammals: Relationships with other constituent corneal structures. Eur. J. Anat..

[158] Wilson S.E. (2020). Bowman’s layer in the cornea– structure and function and regeneration. Exp. Eye Res..

[159] Alarcon I., Kwan L., Yu C., Evans D.J., Fleiszig S.M.J. (2009). Role of the Corneal Epithelial Basement Membrane in Ocular Defense against *Pseudomonas aeruginosa*. Infect. Immun..

[160] Jay L., Brocas A., Singh K., Kieffer J.C., Brunette I., Ozaki T. (2008). Determination of porcine corneal layers with high spatial resolution by simultaneous second and third harmonic generation microscopy. Opt. Express.

[161] Crespo-Moral M., Garcia-Posadas L., Lopez-Garcia A., Diebold Y. (2020). Histological and immunohistochemical characterization of the porcine ocular surface. PLoS ONE.

[162] Batista A., Breunig H.G., Uchugonova A., Morgado A.M., Konig K. (2016). Two-photon spectral fluorescence lifetime and second-harmonic generation imaging of the porcine cornea with a 12-femtosecond laser microscope. J. Biomed. Opt..

[163] Ojeda J.L., Ventosa J.A., Piedra S. (2001). The three-dimensional microanatomy of the rabbit and human cornea. A chemical and mechanical microdissection-SEM approach. J. Anat..

[164] Lai T., Tang S. (2014). Cornea characterization using a combined multiphoton microscopy and optical coherence tomography system. Biomed. Opt. Express.

[165] Hayashi S., Osawa T., Tohyama K. (2002). Comparative observations on corneas, with special reference to Bowman’s layer and Descemet’s membrane in mammals and amphibians. J. Morphol..

[166] Ramphal R., McNiece M.T., Polack F.M. (1981). Adherence Of Pseudomonas-Aeruginosa to the Injured Cornea—A Step In The Pathogenesis Of Corneal Infections. Ann. Ophthalmol..

[167] Vallas V., WienerKronish J.P., Mostov K.E., Fleiszig S.M.J. (1996). Cytotoxic strains of *Pseudomonas aeruginosa* can damage the intact corneal surface. Investig. Ophthalmol. Vis. Sci..

[168] Klotz S.A., Au Y.K., Misra R.P. (1989). A Partial-Thickness Epithelial Defect Increases the Adherence of Pseudomonas-Aeruginosa to the Cornea. Investig. Ophthalmol. Vis. Sci..

[169] Augustin D.K., Heimer S.R., Tam C., Li W.Y., Le Due J.M., Evans D.J., Fleiszig S.M.J. (2011). Role of Defensins in Corneal Epithelial Barrier Function against *Pseudomonas aeruginosa* Traversal. Infect. Immun..

[170] Ubani-Ukoma U., Gibson D., Schultz G., Silva B.O., Chauhan A. (2019). Evaluating the potential of drug eluting contact lenses for treatment of bacterial keratitis using an *ex vivo* corneal model. Int. J. Pharm..

[171] Tam C., Mun J.J., Evans D.J., Fleiszig S.M.J. (2010). The Impact of Inoculation Parameters on the Pathogenesis of Contact Lens-Related Infectious Keratitis. Investig. Ophthalmol. Vis. Sci..

[172] Jian H.J., Yu J.T., Li Y.J., Unnikrishnan B., Huang Y.F., Luo L.J., Ma D.H.K., Harroun S.G., Chang H.T., Lin H.J. (2020). Highly adhesive carbon quantum dots from biogenic amines for prevention of biofilm formation. Chem. Eng. J..

[173] Tang A.H., Caballero A.R., Marquart M.E., Bierdeman M.A., O’Callaghan R.J. (2018). Mechanism of *Pseudomonas aeruginosa* Small Protease (PASP), a Corneal Virulence Factor. Investig. Ophthalmol. Vis. Sci..

[174] Venkatesh M., Barathi V.A., Goh E.T.L., Anggara R., Fazil M., Ng A.J.Y., Harini S., Aung T.T., Fox S.J., Liu S.P. (2017). Antimicrobial Activity and Cell Selectivity of Synthetic and Biosynthetic Cationic Polymers. Antimicrob. Agents Chemother..

[175] Li J., Ma X., Zhao L., Li Y., Zhou Q., Du X. (2020). Extended contact lens wear promotes corneal norepinephrine secretion and *Pseudomonas aeruginosa* infection in mice. Investig. Ophthalmol Vis. Sci..

[176] Kugadas A., Geddes-McAlister J., Guy E., DiGiandomenico A., Sykes D.B., Mansour M.K., Mirchev R., Gadjeva M. (2019). Frontline Science: Employing enzymatic treatment options for management of ocular biofilm-based infections. J. Leukoc. Biol..

[177] Saraswathi P., Aung T., Salleh S., Beuerman R. (2013). An Experimental Model of Biofilm Formation in the Mouse Cornea. Investig. Ophthalmol. Vis. Sci..

[178] Metruccio M.M.E., Evans D.J., Gabriel M.M., Kadurugamuwa J.L., Fleiszig S.M.J. (2016). *Pseudomonas aeruginosa* Outer Membrane Vesicles Triggered by Human Mucosal Fluid and Lysozyme Can Prime Host Tissue Surfaces for Bacterial Adhesion. Front. Microbiol..

[179] Henriksson J.T., McDermott A.M., Bergmanson J.P.G. (2009). Dimensions and Morphology of the Cornea in Three Strains of Mice. Investig. Ophthalmol. Vis. Sci..

[180] Zschaler J., Schlorke D., Arnhold J. (2014). Differences in Innate Immune Response between Man and Mouse. Crit. Rev. Immunol..

[181] Tam C., LeDue J., Mun J.J., Herzmark P., Robey E.A., Evans D.J., Fleiszig S.M.J. (2011). 3D Quantitative Imaging of Unprocessed Live Tissue Reveals Epithelial Defense against Bacterial Adhesion and Subsequent Traversal Requires MyD88. PLoS ONE.

[182] Mun J.J., Tam C., Kowbel D., Hawgood S., Barnett M.J., Evans D.J., Fleiszig S.M.J. (2009). Clearance of *Pseudomonas aeruginosa* from a Healthy Ocular Surface Involves Surfactant Protein D and Is Compromised by Bacterial Elastase in a Murine Null-Infection Model. Infect. Immun..

[183] Wan S.J., Ma S., Evans D.J., Fleiszig S.M.J. (2020). Resistance of the murine cornea to bacterial colonization during experimental dry eye. PLoS ONE.

[184] Yeung J., Gadjeva M., Geddes-McAlister J. (2020). Label-Free Quantitative Proteomics Distinguishes General and Site-Specific Host Responses to *Pseudomonas aeruginosa* Infection at the Ocular Surface. Proteomics.

[185] Sewell A., Dunmire J., Wehmann M., Rowe T., Bouhenni R. (2014). Proteomic analysis of keratitis-associated *Pseudomonas aeruginosa*. Mol. Vis..

[186] Sun Y., Karmakar M., Roy S., Ramadan R.T., Williams S.R., Howell S., Shive C.L., Han Y.P., Stopford C.M., Rietsch A. (2010). TLR4 and TLR5 on Corneal Macrophages Regulate *Pseudomonas aeruginosa* Keratitis by Signaling through MyD88-Dependent and -Independent Pathways. J. Immunol..

[187] Suzuki T., Okamoto S., Oka N., Hayashi N., Gotoh N., Shiraishi A. (2018). Role of pvdE Pyoverdine Synthesis in *Pseudomonas aeruginosa* Keratitis. Cornea.

[188] Clemens L.E., Jaynes J., Lim E., Kolar S.S., Reins R.Y., Baidouri H., Hanlon S., McDermott A.M., Woodburn K.W. (2017). Designed Host Defense Peptides for the Treatment of Bacterial Keratitis. Investig. Ophthalmol. Vis. Sci..

[189] Ni M.J., Tam C., Verma A., Ramphal R., Hawgood S., Evans D.J., Fleiszig S.M.J. (2008). Expression of surfactant protein D in human corneal epithelial cells is upregulated by *Pseudomonas aeruginosa*. FEMS Immunol. Med. Microbiol..

[190] Jiang X.Y., McClellan S.A., Barrett R.P., Zhang Y.F., Foldenauer M.E., Hazlett L.D. (2012). The Role of VIP in Cornea. Investig. Ophthalmol. Vis. Sci..

[191] Barbariga M., Vallone F., Mosca E., Bignami F., Magagnotti C., Fonteyne P., Chiappori F., Milanesi L., Rama P., Andolfo A. (2019). The role of extracellular matrix in mouse and human corneal neovascularization. Sci. Rep..

